# Tailored combination prevention packages and PrEP for young key populations

**DOI:** 10.7448/IAS.18.2.19434

**Published:** 2015-02-26

**Authors:** Audrey Pettifor, Nadia L Nguyen, Connie Celum, Frances M Cowan, Vivian Go, Lisa Hightow-Weidman

**Affiliations:** 1 Department of Epidemiology, University of North Carolina at Chapel Hill, Chapel Hill, NC, USA; 2 School of Public Health, University of the Witwatersrand, Johannesburg, South Africa; 3 International Clinical Research Center, University of Washington, Seattle, WA, USA; 4 Centre for Sexual Health and HIV/AIDS Research (CeSHHAR) Zimbabwe, Harare, Zimbabwe; 5 Department of Infection and Population Health, University College London, London, United Kingdom; 6 Department of Health Behavior, University of North Carolina at Chapel Hill, Chapel Hill, NC, USA; 7 Department of Infectious Diseases, University of North Carolina at Chapel Hill, Chapel Hill, NC, USA

**Keywords:** HIV, key populations, combination prevention, pre exposure prophylaxis

## Abstract

**Introduction:**

Young key populations, defined in this article as men who have sex with men, transgender persons, people who sell sex and people who inject drugs, are at particularly high risk for HIV. Due to the often marginalized and sometimes criminalized status of young people who identify as members of key populations, there is a need for HIV prevention packages that account for the unique and challenging circumstances they face. Pre-exposure prophylaxis (PrEP) is likely to become an important element of combination prevention for many young key populations.

**Objective:**

In this paper, we discuss important challenges to HIV prevention among young key populations, identify key components of a tailored combination prevention package for this population and examine the role of PrEP in these prevention packages.

**Methods:**

We conducted a comprehensive review of the evidence to date on prevention strategies, challenges to prevention and combination prevention packages for young key populations. We focused specifically on the role of PrEP in these prevention packages and on young people under the age of 24, and 18 in particular.

**Results and discussion:**

Combination prevention packages that include effective, acceptable and scalable behavioural, structural and biologic interventions are needed for all key populations to prevent new HIV infections. Interventions in these packages should meaningfully involve beneficiaries in the design and implementation of the intervention, and take into account the context in which the intervention is being delivered to thoughtfully address issues of stigma and discrimination. These interventions will likely be most effective if implemented in conjunction with strategies to facilitate an enabling environment, including increasing access to HIV testing and health services for PrEP and other prevention strategies, decriminalizing key populations’ practices, increasing access to prevention and care, reducing stigma and discrimination, and fostering community empowerment. PrEP could offer a highly effective, time-limited primary prevention for young key populations if it is implemented in combination with other programs to increase access to health services and encourage the reliable use of PrEP while at risk of HIV exposure.

**Conclusions:**

Reductions in HIV incidence will only be achieved through the implementation of combinations of interventions that include biomedical and behavioural interventions, as well as components that address social, economic and other structural factors that influence HIV prevention and transmission.

## Introduction

Globally young people face a high burden of HIV infection. It is estimated that 39% of new infections occur among adolescents annually, and despite global declines in HIV mortality among adults [1], HIV-related deaths among young people increased by 50% between 2005 and 2012 [2]. Key populations, defined here as men who have sex with men (MSM), transgender persons, sex workers and people who inject drugs (PWID), experience a high burden of HIV infection and incidence rates in both concentrated and generalized epidemic settings. It is estimated that up to 50% of new infections occur among key populations annually [2].

Young people (which we define as persons between 10 and 24) who fall under the umbrella term “key population” are at particularly high risk for HIV and may engage in overlapping risk behaviours, such as injecting drugs and selling sex. While data are scarce on the size of adolescent key populations (defined as ages 10–19 years), in areas of the world where the epidemic is concentrated among key populations, adolescents clearly face an increased burden. It is estimated that 95% of new infections among adolescents in Asia are among key populations (PWID, MSM and sex workers) [3] and that 70% of all individuals who inject drugs are under the age of 25 [4]. A number of studies have documented that many individuals who engage in sex work or injection drug use began before the age of 18 [5, 6]. Among MSM globally, infection rates continue to increase in many settings [7]. HIV incidence data from the United States highlight the crisis of HIV among young MSM (YMSM); from 2008 to 2011, HIV incidence for YMSM aged 13–24 years increased 26% [8]. Due to this increased risk, multiple programmatic calls have been issued to refocus prevention efforts on adolescent and youth key populations. Reductions in HIV incidence will only be achieved through the implementation of combinations of interventions that include biomedical and behavioural interventions, as well as components that address social, economic and other structural factors that influence HIV prevention and transmission [9–15]. Antiretroviral-based prevention, specifically pre-exposure prophylaxis (PrEP), is one biomedical prevention approach that has recently shown great promise in reducing risk of HIV acquisition [16–20]. However, its effectiveness in some adolescent key populations remains unclear.

In this article, we review the current evidence on prevention strategies for young key populations and specific challenges to HIV prevention unique to young key populations, describe what an effective and tailored combination prevention package would look like for young key populations and discuss the role of PrEP as a potential component of that prevention package.

## Methods

We conducted a comprehensive review of the evidence to date on prevention strategies, challenges to prevention and combination prevention packages for young key populations. We focused specifically on the role of PrEP in these prevention packages for young key populations under the age of 24, and under the age of 18 in particular. We examined the published literature by searching PubMED using the following search terms: PrEP, MSM, IDU, PWID, Sex work and HIV prevention. We also examined the works cited of published articles. We identified ongoing studies of PrEP by examining the AVAC database of ongoing and planned PrEP evaluation studies, conference abstracts and the NIH Research Portfolio Online Reporting Tools (RePORT). We did not utilize any exclusion criteria; however, we focused our search on studies or evaluations of PrEP among young [18–24], key populations (MSM, PWID and people who sell sex).

## Results and discussion

### Challenges to HIV prevention among young key populations

Young key populations are at increased risk of HIV infection compared to adults due to cognitive, contextual and structural factors that increase their vulnerability to peer pressure, manipulation and exploitation or abuse by older people [21]. At the same time, young key populations are a heterogeneous group and the risk factors for HIV differ across young key populations and vary by age and setting.

### Young PWID

Young PWID face a number of challenges to HIV prevention. PWID aged 18–29 are more likely to inject daily than other age groups [22], more likely to share syringes than other age groups [22], less likely to use harm reduction and treatment services, more likely to be reliant on older people for access to drugs and injecting equipment, more likely to obtain needles from unofficial sources, and less informed about risks and their rights [23]. Female PWIDs frequently experience violence from intimate partners, police and sex trade clients [24], as well as homelessness [25] and psychiatric co-morbidities [26], which may act synergistically, increasing their risk for HIV infection [23]. Young female PWID in particular may face unique risks for HIV, including mental health disorders [27], and high suicide risk [28]. In addition, young female injectors have higher injecting risk behaviours compared to young male injectors, including multiple sex partners [29] and co-infection with HIV and HCV [30].

Despite existing evidence-based prevention tools for PWID populations, including opioid substitution therapy (OST) [31–34], needle and syringe exchange programs (NSP) [31, 35, 36] and HIV testing and counselling (HTC) [31, 37], the epidemic among PWID continues to accelerate in many settings [38] while the proportion of youth who are PWID continues to increase [39].

### Young MSM and transgender persons

Young MSM experience multiple life stressors and high levels of victimization based on sexual identity that can lead to engagement in higher sexual and drug use activities, and also make practicing HIV prevention strategies challenging [40–42]. Compared to their heterosexual peers, YMSM have been found to have an increased risk of depressive symptoms, anxiety disorders, suicidal ideation and attempts, and PTSD [43–45]. Some YMSM may experience homelessness or unstable housing as a result of being driven out of their family homes. Further, YMSM face additional social challenges in developing a positive self-identity due to stigmatization, discrimination and homophobia. The challenges that place YMSM, and in particular YMSM of colour, at risk for HIV infection also impact their awareness, access to, and adherence to prevention services, including PrEP [46–50]. For instance, despite routine testing recommendations, MSM who are younger (<25 years), black, and/or have low income are less likely to test or be aware that they are HIV-infected [51–55]. These challenges are magnified in areas where homosexuality is criminalized.

Young transgender women are also at extremely high risk of HIV infection due to multiple concurrent risk factors, including substance use, sex work, depression, unstable housing, discrimination, violence and victimization [56–59]. Limited access to gender-sensitive health services can also interfere with HIV prevention efforts.

### Young people who sell sex

Young people who sell sex also face challenges that put them at greater risk of HIV when compared to adult sex workers. These include a heightened risk of physical and sexual violence by clients and law enforcement agents [60–63]. As a result of exploitation by adults, young people who sell sex may lack control over the frequency and location of where they sell sex, and may be more likely to work on the streets than adults [64–67]. Young people who have been orphaned or abandoned by their family face social and economic marginalization; consequently, in many parts of the world, children living on the street sell sex as a survival strategy [68–70]. In addition, young people who sell sex use condoms less consistently than adult sex workers due to lack of access to condoms, poor negotiating skills and limited knowledge of issues related to sexual and reproductive health. Young people who sell sex also face stigma and discrimination, which not only affects their ability to access services but may also lead to low self-worth and self-stigmatization [71]. Young people who sell sex may also be more difficult to reach with services because initiation into sex work may be gradual and thus they may not recognize themselves to be at risk.

### Legal and structural barriers to HIV prevention

Across all young key populations, parental permission laws in many settings poses an additional challenge for delivering effective prevention packages to this age group because they prevent minors from accessing prevention and care services without the involvement of a parent. A recent survey by UNAIDS found that over 33 countries in Africa have age based criteria for HTC [72]. In addition, young people often do not seek health services due to stigma associated with youth attending HIV prevention services, and lack of youth friendliness and confidentiality in many health settings [73]. These structural barriers are even greater for young key populations because their behaviours are stigmatized and illegal in many settings, resulting in discrimination, marginalization, possible legal consequences (such as imprisonment) and fear of punishment [3]. In countries where homosexuality is illegal, YMSM who fear being outed by health workers may delay care. Laws that classify sex work among people who are under 18 as sexual exploitation (designed to protect minors involved in the sex industry), may have the unintended consequence of encouraging young women who sell sex to deny involvement or avoid health services because of fear of being sent to state institutions or suffering abuse and harassment by law enforcement [74–80] . Laws requiring parental permission for prevention services also fail to recognize that many adolescents engaged in injecting drug use or selling sex do not live with family or may be orphans.

### Combination prevention packages for young key populations

Combination prevention packages that include effective, acceptable and scalable behavioural, structural and biologic interventions are needed for all key populations in order to have the greatest impact on preventing new infections. This is supported by mathematical modelling which has found that existing structural and behavioural prevention approaches for key populations could be further strengthened by combining them with newer biomedical prevention interventions, such as PrEP [9–15] . Combination prevention packages should aim to achieve high coverage of HIV testing and knowledge of HIV serostatus, parsimony in selecting evidence-based interventions, synergy such that the effect of a combination of interventions is at least the sum of the parts, if not greater, and intervention coverage, which is a function of access to, utilization of, and high retention (see Table 1) [81]. Based on recent guidelines from the WHO for HIV prevention, diagnosis, treatment and care for key populations, combination prevention packages should also include the key health care sector interventions as summarized in Table 2 and strive to create an enabling environment. Among key populations, interventions that meaningfully involve beneficiaries in the design and implementation of the intervention, and take into account the context in which the intervention is being delivered to thoughtfully address issues of stigma and discrimination are most likely to be most effective.

**Figure 1:**
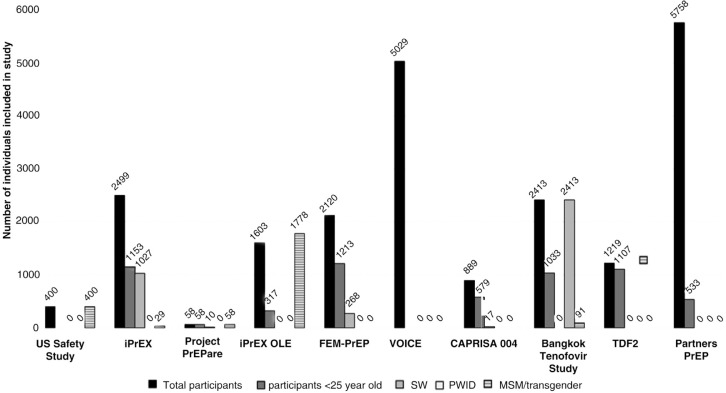
Representation of key populations and young people included in completed PrEP studies. Bangkok and TDF2 participants in “participants <25 category” includes participants under 30 years of age. Number of participants in “participants <25” unknown for US Safety study and VOICE study.

**Table 1:** Principles of combination prevention

Principle	Details
1. High coverage of HIV testing and knowledge of HIV serostatus	HIV testing is the “gateway” to both the HIV treatment and prevention cascades; HIV testing programs need to facilitate linkages to care and prevention
2. Parsimony in selecting evidence-based interventions	Scale, coverage, affordability and impact could be compromised with more complex combination packages
3. Pilot work to determine the acceptability and feasibility of scaling these interventions	Achieve high coverage by prioritizing the subset of the population most at risk of HIV transmission or acquisition
4. Synergy such that the effect of a combination of interventions is at least the sum of the parts, if not greater	Delivering non-overlapping and complimentary interventions to reduce HIV infectiousness and susceptibility
5. Intervention coverage	A function of access to the interventions, willingness of persons prioritized based on risk to utilize the interventions, high retention in the prevention/treatment cascade

**Table 2:** Key components of a comprehensive prevention package

The WHO comprehensive package for prevention			
*Essential health sector interventions*	*IDU*	*Sex workers*	*MSM*
1. Comprehensive condom and lubrication programming	✓	✓	✓
2. Harm reduction interventions for substance use	Needle and syringe programs and opioid substitution therapy		
3. Behavioural interventions	✓	✓	✓
4. HIV testing and counselling	✓	✓	✓
5. HIV treatment and care	✓	✓	✓
6. Sexual and reproductive health interventions	✓	✓	✓
7. Prevention and management of co-infections and other co-morbidities	Viral hepatitis, tuberculosis and mental health conditions	Mental health; substance use	Mental health; substance use
*Essential strategies for an enabling environment*	*Examples*		
1. Supportive legislation, policy and financial commitment	Decriminalization of NSP and OST programs		Social Protection; Decriminalization
2. Addressing stigma and discrimination	✓	✓	✓
3. Community empowerment		✓	
4. Addressing violence against people from key populations	✓	✓	✓
*PrEP plus adherence support*	✓^a^	✓^a^	✓

a Note that the WHO has currently only issued a strong recommendation for PrEP use among MSM. The WHO has made no recommendations regarding PrEP among PWIDs and sex workers but has called for PrEP demonstration projects to assess how to implement PrEP as part of comprehensive risk reduction services in these populations. OST, opioid substitution therapy; NSP, needle and syringe exchange programs.

### PrEP as a potential component of combination prevention packages

PrEP has recently emerged as a promising biomedical intervention to prevent HIV infection [16–20] (see Table 3). For adolescent and young key populations, PrEP could offer a highly effective, time-limited primary prevention if they can access health services and are motivated to use PrEP while at risk of HIV exposure. Although no PrEP efficacy trials completed to date exclusively recruited adolescents and young persons, all the trials included persons between ages 18 and 24 (see Table 4 and Figure 1). Nonetheless, young key population face unique challenges that may influence their willingness to use and adhere to PrEP. Addressing these challenges will be key to the success of PrEP as an intervention strategy in this vulnerable population.

**Table 3:** Completed PrEP studies among key populations and young people

Trial name and location	Number enrolled	Median age (Range)	Study population	Young people/key populations, N (%)	Design and intervention	Percent relative reduction in HIV incidence (95% CI; *p*-value)	Adherence
The Bangkok Tenofovir Study [18] Thailand	2413	31 (20–60)	PWID	Under 30 years old: 1033 (43%) PWID: 2413 (100%) MSM: 91 (5%)	Randomized controlled trial – TDF – Placebo	48.9% (95% CI: 9.6, 72.2%; *p*=0.01)	Drug diaries: 83.8% DOT: 86.9% Blood plasma: TDF detected in 66% in TDF group (overall); TDF detected in 39% among participants who seroconverted; TDF detected in 67% among participants who did not seroconvert
CAPRISA 004 [20] KwaZulu Natal, South Africa	889	23.9 (mean) (18–40)	Women	Under 25 years old: 579 (65.1%) SW: 17 (1.9%)	Randomized controlled trial – TDF vaginal gel (BAT24) – Placebo	39% (95% CI: 6, 60%; *p*=0.017)	Monthly (applicator) count divided by number of sex acts that month: 72.20% (all participants); 61.3% among women who did not seroconvert; 59.2% among women who did seroconvert Blood plasma: 50.5%
FEM-PrEP [82] Kenya, South Africa, Tanzania	2120	23 (18–35)	Women	Under 25: 1213 (57.2%) SW: 268 (12.6%)	Randomized controlled trial – TVD – Placebo	Stopped for futility	Self-report: 95% Pill count: 88% Blood plasma: TVD detected in 26% at beginning of infection window, 21% at end of window and 15% at both visits among women who seroconverted; TVD detected in 35% at beginning of the infection window, 37% of women at end of the window and 24% at both visits among women who did not seroconvert
iPrEx [16] US, Brazil, Peru, Ecuador, Thailand, South Africa	2499	27 (mean) (18–67)	MSM TGW	Under 25: 1153 (46%) TGW: 29 (1%) SW: 1027 (41%)	Randomized controlled trial – TVD – Placebo	44% (95% CI: 15, 63%; *p*=0.005) No significant difference across age	Self-reported pill use: 95% Pill count of unused study product: 89–95% Blood plasma: TVD detected in 9% among participants who seroconverted; TVD was detected in 51% among participants who did not seroconvert
iPrEx OLE [83] Peru, Ecuador, US, Brazil, Thailand, South Africa	1603	28 (mean) (18–40 +)	MSM TGW	Under 25 years old: 317 (20%) MSM: 1603 (100%) TGW: 175 (11%)	Open-label extension – 1225 (76%) received TDF	49% (95% CI: −1, 74%)	Blood plasma: 71% (week 4, 8, or 12)
Partners PrEP [17] Kenya, Uganda	4758	Women: 33 Men: 34 (18–65)	Sero-discordent couples	Under 25: 533 (11%)	Phase 3 study extension/ rollover Randomized controlled trial – TDF – TVD – Placebo	TDF: 67% (95% CI: 44, 81%; *p*<0.001) TVD: 75% (95% CI: 55, 87%; *p*<0.001) No significant difference between women <25 and ≥25	Bottle count: 98% Pill count: 97% Blood plasma: TDF/TVD detected in 31% among participants who seroconverted; TDF/TVD detected in 82% among participants who did not seroconvert
Project PrEPare (ATN 082) [84] US (Baltimore, Boston, Chicago, Denver, Detroit, Houston, Los Angeles, Memphis, Miami, New Orleans, Philadelphia, Tampa)	68	20 (18–22)	Young MSM (focus on MSM of colour)	Under 25: 58 (100%) MSM: 58 (100%) SW: 10 (17.24%)	Feasibility and acceptability study – 3MV (Many Men, Many Voices behavioural HIV intervention) alone – 3MV with TVD (*N*=20) – 3MV with placebo	n/a - Feasibility and acceptability study	Self-reported: 62% (range 43–83%) Blood plasma: 63.2% (week 4), 20% (week 24)
TDF2 (CDC 494) [19] Botswana	1219	25 (18–39)	Men and women (mostly young)	Under 21: 25 (2%) Age 21–29: 1082 (89%)	Randomized controlled trial – TVD – Placebo	62.2% (95% CI: 21.5, 83.4%; *p*=0.03)	Pill count: 84.2% (TVG group) Self-reported 3 days prior: 94.4% (TVD group) Blood plasma: TVD detected in 50% among participants who seroconverted; TVD detected in 81% among participants who did not seroconvert
US Safety study (CDC 4323) [85] US (San Francisco, Atlanta, Boston)	400	39 (18–60)	MSM	Under 25: Unknown MSM: 400 (100%)	Phase II safety study – TDF upon enrolment – Placebo upon enrolment – TDF 9 months after enrolment – Placebo 9 months after enrolment	n/a - Safety study	
VOICE (MTN 003) [86] Uganda, Zimbabwe, South Africa	5029	25.3 (mean) (18–45)	Women	Under 25: unknown	Phase IIb (proof of concept) trial – TVD – TDF – TDF vaginal gel – Placebo (pill) – Placebo (gel)	Stopped for futility	Self-report and pill/applicator count: ~90% Blood plasma: TVD detected in 29% in TVD group (overall); TVD detected in 21% in TVD group (≤25, single); TVD detected in 54% in TVD group (>25, married); TDF detected in 28% in oral TDF group; TDF detected in 23% in TDF gel group
Willingness of PWID to use PrEP in Ukraine [87] Ukraine	128	(16–40+)	PWID	Under 25/PWID: 22 (17% of PWIDs)	Willingness to accept and use PrEP	n/a 53% stated they would “definitely” be willing to use PrEP (based on a 4-point Likert scale) 32.6% stated they would “probably” be willing to use PrEP	n/a

MSM, men who have sex with men; TWG, transgender women; SW, sex workers; PWID, people who inject drugs; TDF, tenofovir; TVD, emtricitabine/tenofovir (FTC/TDF).

**Table 4:** Overview of completed and ongoing PrEP studies targeting young people and key populations, by population and PrEP type/mode of delivery

Target population	Oral PrEP and combination prevention	Dosing/alternative formulations of oral PrEP	Topical PrEP^c^
Under 18 years old	CHAMPS-SA Plus Pills^a^ FACTS 002^a^ Project PrEPare (ATN 113)^a^		
MSM/TGW	California Collaborative Treatment Group Consortium/ALERT (CCTG 593) DemoPrEP The Demo Project (NIAID) East Bay Consortium/CRUSH^b^ HPTN 073 Los Angeles County PATH PrEP Demo Project LVCT and SWOP Project PrEPare (ATN 110)^b^ Project PrEPare (ATN 113)^a^ PROUD Sibanye Health Project SPARK Project NYC Sustainable Health Center Implementation PrEP Pilot Study (SHIPP) (CDC Foundation) VicPrEP Demonstration Project	ADAPT (HPTN 067) IPERGAY NEXT-PREP (HPTN 069/ACTG 5305)	MTN 017
SW	Durbar (DMSC) and Ashodaya Samithi LVCT and SWOP SAPPH-IRe TAPS: Expanded use of ART for treatment and prevention for female sex workers in South Africa Wits Reproductive Health and HIV Institute		
PWID	Bangkok Tenofovir Study Open-Label Extension Sustainable Health Center Implementation PrEP Pilot Study (SHIPP) (CDC Foundation)		

a Participants 18 and younger.

b participants 24 and younger.

c note that there are other efficacy trials of topical PrEP (e.g., FACTS 001, ASPIRE, Ring Study) but they do not exclusively target young people or key populations.

Adherence to medications is known to be a significant challenge for young people, [88–91] and thus adherence to PrEP must be an important focus of any intervention providing PrEP to this population [92]. Across all the PrEP trials, there is robust evidence that PrEP has high effectiveness, but this effectiveness is highly dependent on adherence [11, 93]. Sub-analyses of existing trial data suggest that younger and unmarried participants as well as those with highest behavioural risk were the least likely to adhere to PrEP [17, 20, 94]. These results are in line with evidence from other medical conditions, which have found that between 10 and 90% of adolescents demonstrate inadequate adherence to therapy, and those least likely to adhere are the most vulnerable psychosocially [89, 95, 96]. Notably, all the PrEP trials had a subset of persons who had consistent and sustained use of PrEP, which ranged from 30% in the VOICE [94] and FEM-PrEP [82] trials to 80% in the Partners PrEP Study [17].

Concerns about adherence to PrEP and subsequent drug-resistance are particularly strong for PWID [97], whose barriers to antiretroviral therapy (ART) adherence include interruptions in care due to low social support, incarceration, and compulsory detoxification and detention [98]. At the same time, a recent meta-analysis revealed that PWID had comparable rates of ART adherence to non-drug using populations [98] suggesting that these concerns may be unfounded.

There are limited data on adherence to ART among persons who sell sex [99, 100]. Some reports suggest that persons who sell sex may be poorly adherent due to their social instability, increased mobility and police harassment, but there are also data suggesting that persons who sell sex can adhere if properly supported. However, while we can learn from studies on ART adherence, the barriers to adherence may be quite different among HIV-negative PrEP users [101]. There is a critical need to understand the reasons for poor PrEP adherence among young women, including sex workers [102]. Several upcoming studies and demonstration projects are examining the impact of different adherence counselling programs and delivery mechanisms to improve PrEP adherence among participants (see Table 5, Supplementary files).

The differential uptake and sustained use among populations enrolled in placebo-controlled PrEP efficacy trials in part reflects population differences in terms of levels of uncertainty and ambivalence about using antiretrovirals for prevention, risk perception, concerns about side effects, stigma, reactions of others, partner support, participation in a placebo-controlled trial to obtain access to health care and other services, and concerns about randomization to placebo or a product of uncertain efficacy [103–105] . Uptake and adherence among participants in clinical trials who are randomized to placebo or active product and counselled about unknown efficacy may not predict uptake and adherence among at risk participants who are offered open-label product and counselled about known efficacy and the importance of adherence. Encouragingly, two studies of daily and intermittent oral PrEP among MSM were recently stopped early due to high effectiveness: 1) the immediate daily oral PrEP arm in the United Kingdom compared to the delayed PrEP arm in the PROUD study [106], and 2) the intermittent, event-driven dosing of Truvada arm compared to the placebo arm in France and Quebec in the IPERGAY study [107]. The high effectiveness demonstrated early in these studies indicate that adherence to oral PrEP among MSM is high in the context of known efficacy even when delivered with less intensive adherence counselling.

In addition, new studies and ongoing demonstration projects are examining new PrEP formulations and coitally-dependent pill/gel-schedules, which may simplify and improve adherence (see Table 5, Supplementary files). Long acting injectable and slow release delivery mechanisms (for example, using a vaginal ring) are currently being evaluated for efficacy and may be available for more real world evaluation within the next 1–3 years. Antiretrovirals (including dapivirine and tenofovir) are being formulated in sustained release vaginal rings combined with levonorgestrol for contraception (multi-purpose technologies), which may further enhance uptake and adherence for young women [108, 109]. These new PrEP delivery mechanisms are likely to be highly applicable to adolescent key populations as they do not require daily pill taking which may prove difficult for some adolescents, particularly those with unpredictable lives, unstable living situations, and/or mental health or substance use issues.

In sum, the efficacy of oral TDF and FTC/TDF has been demonstrated across multiple studies, and demonstration projects are currently evaluating strategies to improve access to, uptake of and adherence to PrEP in key populations (see Table 4 and Table 5 in Supplementary files). PrEP has great promise if integrated into a combination prevention package that provides support for the structural and behavioural barriers to this innovative biomedical prevention strategy, including accessing health care, assessing one's risk and motivation for prevention, and developing adherence habits. Below we will highlight what an ideal combination package for young key populations might look like and the potential role of PrEP within such a package.

### Combination prevention for MSM and transgender persons

An ideal combination prevention package for YMSM and young transgendered persons would include effective interventions to address behavioural risk factors, PrEP uptake and adherence support as well as addressing structural barriers to prevention (including criminalization, stigma, discrimination and homophobia). High rates of mobile phone ownership and technology use among youth provide a unique platform to deliver tailored, engaging HIV health promotion interventions to YMSM and young transgendered persons [110–112]. For example, a combination prevention app could include features to 1) increase HIV testing (e.g. provide youth with access to nearby HIV testing locations or facilitate ordering of home HIV tests); 2) help YMSM and young transgendered persons successfully access and adhere to PrEP (e.g. tracking of pill taking, side effects, pharmacy refill information); and 3) enhance patient provider interactions to ensure timely and comprehensive follow-up (e.g. symptom tracker to document any symptoms of acute HIV infection, reminders for HIV and other testing). However, to date behavioural and structural HIV prevention interventions designed specifically for YMSM and young transgendered persons are severely lacking. A recent review of primary HIV prevention interventions for adolescents/young adults found that of the 92 articles reviewed, only three unique interventions were specifically tailored to the needs of gay/bisexual male adolescents and young adults [113].

Young transgender women may require a fairly different package of combination HIV prevention interventions than young MSM. Although they may share some similar structural and social barriers, they face unique challenges, including those related to transitioning, gender discrimination, transphobia and violence [114]. A recent review has highlighted the lack of evidence-based interventions for transgender populations and the need to understand differences between MSM and transgender populations and the heterogeneity within the group so that prevention and care can be implemented more effectively [115].

Currently two studies have been conducted that have offered PrEP to younger MSM (Project PrEPare and iPrEx OLE), while only one study has included transgender persons (iPrEx OLE) [83, 84] (see Table 3). Transgender persons have been largely underrepresented in biomedical and behavioural prevention trials and more work is needed to determine the ideal set of interventions in a combination prevention package for this population [114]. In contrast, in the two years since the FDA approved Truvada for PrEP, there is growing momentum in policy related to PrEP for MSM. CDC guidance in 2014 made PrEP a central part of US prevention efforts [116], and it has been featured as one of the three key components of the New York state response to reduce new HIV infections [117]. In 2014 WHO issued guidelines for PrEP implementation which focused on MSM [118].

Project PrEPare was a pilot study conducted in the US that used a randomized 3-arm design to compare an efficacious behavioural HIV prevention intervention (Many Men, Many Voices—3 MV) alone with 3 MV combined with PrEP (tenofovir/emtricitabine), and 3 MV combined with placebo [84]. For the purposes of this trial, the 3 MV intervention was adapted for use with youth groups of mixed racial and ethnic identities. Sixty-eight youth (mean age=19.97 years; 53% African American, 40% Latino) were enrolled, 58 were randomized, 20 received PrEP and no one under the age of 18 was included [84]. Although acceptability (size of the FTC/TDF pill) was an issue for some men, the study found that 62% had tenofovir detected in plasma samples, which is an encouraging finding in this age group, and likely could be improved with an adherence support intervention during PrEP use. Future PrEP demonstration projects among YMSM should focus on acceptability, motivation and adherence support for men who are motivated to take PrEP.

To date some of the structural barriers to uptake of PrEP among YMSM have included cost of the medication and the comprehensive services required for those on PrEP, and limited access to primary care. Providers may also be not offering PrEP to those most in need. To improve uptake of PrEP, we recommend more fully integrating the provision of PrEP into sexually transmitted infection (STI) services and educating health care providers about the efficacy of PrEP and strategies for providing culturally competent and non-judgmental care for young key populations. We anticipate that the provider reluctance to prescribe PrEP will decrease in the wake of the PROUD and IPERGAY results, which indicate that MSM were able to make informed decisions about their risks and need for PrEP and adhere sufficiently to obtain substantial prevention benefits.

### Combination prevention for young people who sell sex

Combination prevention for HIV in young people who sell sex should include behavioural, structural and biomedical interventions. Community empowerment, condom promotion, HTC with linkage to treatment and care services, STI treatment and health education have been shown to be effective interventions for sex workers, but they have not been taken to scale or adequately resourced in most parts of the world [9].

To be effective, interventions targeting young people who sell sex must address their specific needs and the unique barriers they face to accessing programs for adult sex workers. For example, young sex workers may not perceive HIV prevention programs to be relevant to them, and may face competition from adult sex workers, who act as gatekeepers to sex worker HIV prevention programs. Tailored programs for younger women also need to encompass interventions that address issues of social protection which can be implemented as required on a case by case basis. Given that the majority of sex workers who acquire HIV are infected early in their career, programs need to have a strategy for identifying young people shortly after they start selling sex, and to facilitate their timely engagement with prevention services [119].

Access to prevention services is also often hampered by the legal and policy environment. UNAIDS defines sex workers as “people who receive money or goods in exchange for sexual services, either regularly or occasionally”[120], while the Convention on the Rights of the Child considers anyone selling sex under age 18 years to be sexually exploited [71]. Governments have a legal obligation to protect those under 18 from sexual exploitation and this obligation frequently results in a “raid and rescue” response to HIV prevention which perversely results in increased vulnerability and decreased access to HIV prevention services [121]. Criminalization of sex work in many settings results in young people who sell sex being afraid to seek services because of fear of arrest or imprisonment. Some countries have mandatory reporting laws for people under 18 selling sex which put health care providers in direct conflict with their responsibility to provide confidential care [75].

Although there are examples of small scale HIV prevention programs targeting young people who sell sex, these existing approaches need to be scaled up more widely and evaluated to realize improvements in HIV prevention and sexual and reproductive health among this group. For example the SHARPER project in Accra, Ghana effectively uses young peer educators who are paired with older women in the community “peer protectors”. The program focuses on health education, skills building, assisting with linkage to services and violence prevention [122]. In the Philippines, the River of Life Initiative works with young MSM who sell sex and uses peer to peer outreach to contact these hard to reach young men [123].

To date, there have been no completed trials of PrEP conducted specifically among sex workers (although two of the six trials demonstrating efficacy included sex worker participants, see Table 3). However, when the number needed to treat (NNT) to avert one HIV infection was estimated among sub-sets of women in the Partners PrEP trial, the NNT was lowest among women under 30 years and women who reported multiple high-risk behaviours. These findings suggest that the number of young women who sell sex that would need to access PrEP to prevent one infection is likely to be favourable PrEP can be safely and effectively implemented [124].

We know already that PrEP for young people who sell sex should not be considered as a stand-alone intervention, but will need to be implemented within a comprehensive package of interventions that strengthen community cohesion (such as those described in the examples above) alongside behavioural/technological approaches to build individual agency, self-efficacy and skills. The intervention components will need to be relevant to, and address the specific concerns of, young people who sell sex and be implemented in conjunction with them. It is likely that the exact form and delivery of comprehensive prevention will be context and culture specific. The next step will be to use formative research to develop and test comprehensive prevention packages for young people who sell sex, which can then be rigorously evaluated as they are scaled up using impact evaluation.

### People who inject drugs

UNAIDS has identified nine interventions considered essential to prevent HIV among IDUs. These interventions consist of needle syringe programs, opioid substitution treatment, HTC, ART, STI treatment, condom distribution, information and education campaigns, vaccination and treatment of viral hepatitis, and prevention and treatment of tuberculosis [29]. In this context, PrEP is a promising addition to the existing cadre of evidence-based interventions especially given that tenofovir does not alter the pharmacokinetics or pharmacodynamics of methadone or benprenorphine [125].

While evidence on PrEP among key populations is growing, studies with empirical data collection among PWID are limited to one PrEP efficacy trial among PWID (see Table 3). The Bangkok Tenofovir Study which was a phase III randomized double-blind placebo-controlled trial to evaluate the efficacy of PrEP with daily oral tenofovir on HIV infections in PWID [18].

Despite the promising results of the Bangkok Tenofovir Study, some have questioned whether PrEP provided protection against parenteral HIV exposure, given the low and declining incidence of reported injection and needle sharing behaviours during the trial. Although it is not possible to distinguish between the proportion of infections in the Bangkok Tenofovir Study that were attributable to parenteral versus sexual transmission [126], the key finding was the halving of HIV incidence in the PrEP arm. This is a generalizable result for HIV protection for PWIDs given that many PWID populations are at risk of HIV through both parenteral and sexual exposure. Notably, the majority of study participants were on methadone maintenance and in both arms, and injecting risk behaviours, including injecting and needle sharing decreased dramatically over three years of follow-up, suggesting that parenteral transmission may have only contributed a small proportion of the incidence. Thus, the Bangkok Tenofovir Study demonstrates that daily oral tenofovir significantly reduces HIV transmission among PWID in the context of opiate substitution therapy, and thus is a demonstration of effective combination prevention for PWID.

Several challenges remain for implementing PrEP among PWID outside a research setting. In many settings, injecting drug use is highly stigmatized, and PWID-specific HIV prevention interventions do not have adequate governmental or public support [127, 128] leading to suboptimal implementation of known highly effective prevention methods [23, 129]. Until these evidence-based intervention components including NSP, OST and HTC are successfully implemented, the role of PrEP may be limited. A recent systematic review of barriers to treatment among PWID [93] found that structural barriers, including incarceration, inadequate housing, and lack of a legal income [130, 131], were more common than individual-level barriers to accessing HIV treatment and care. In order for PrEP to be successfully implemented, a supportive political, social and environmental platform is imperative.

PrEP is not a replacement for other evidence-based programs. Rather, PrEP should be considered as part of a combination prevention package that includes other proven prevention strategies such as OST, NSP and HTC [23, 31, 34, 129, 132]. A package that integrates and provides PrEP into drug treatment programs and pharmacies and HTC clinics where there is the ability to frequently perform HIV testing and create linkages to providers to monitor patients would be ideal. In addition, it will be important to package PrEP with interventions that have been shown to increase adherence among PWID, particularly when targeting adolescent PWID, such as directly observed therapy and methadone maintenance therapy. Sub-populations of adolescent PWID such as young injecting initiates are more likely to be homeless [133] and engage in a range of risk behaviours including hazardous alcohol use, cocaine use, crystal methamphetamine use [133], unprotected sex [134, 135] and survival sex [133]. The concurrent high-risk behaviour and lack of effective treatments for cocaine or methamphetamine dependency underscore the importance of PrEP in this population [129] while at the same time highlighting their specific adherence challenges related to alcohol use [136, 137] and homelessness [138, 139]. Behavioural strategies that are part of a comprehensive approach for young people should encourage the delay of sexual debut, emphasize a reduction in the number of sexual partners and encourage the use of voluntary HTC services without concern for penalization. Further research is still needed to identify the most effective combination of interventions for PWID with an understanding that packages will need to be tailored for specific settings and sub-populations of drug users, such as adolescent and young PWID.

## Conclusions

Effective yet scalable combination packages are needed for young key populations. To date, adolescents generally, and adolescent key populations specifically, have not been included in studies of biomedical and combination prevention due to regulatory and parental permission related issues [140]. In an era of constrained resources, we need to identify intervention components that are most effective at addressing the key issues for the target population. In many settings, young key populations are at highest risk of infection. While the key populations highlighted in this paper face unique risks for HIV, they also share many important challenges to prevention, including stigma, marginalization, discrimination and, in some cases, criminalization. It is critical that we address these structural risk factors when developing prevention packages for these populations.

With regard to PrEP as part of any combination prevention package, the World Health Organization strongly recommends the use of oral PrEP among MSM based on the evidence that PrEP works in this population and is safe if taken as prescribed [21]. Improving knowledge about PrEP, and uptake of and adherence to this intervention among YMSM who have an incredibly high incidence of infection is a priority. For young PWID, expansion of harm reduction, specifically needle and syringe programs, and OST is a critical first step to creating an environment conducive to PrEP. Among sex workers, although no PrEP trials to date have specifically targeted sex workers, in particular young sex workers, PrEP has shown to be efficacious in trials that included individuals who report trading sex for money or housing. Structural impediments, including policy/law, stigma and access to health service will not be addressed by efficacy or behavioural trials, thus major policy, educational and advocacy work will be needed along with the prevention components discussed here. For all of these populations, there is a need to address critical enablers to access to HIV testing and health services for PrEP and other prevention strategies, including decriminalization of key populations’ practices, improved access to prevention and care, a reduction in stigma and discrimination, and community empowerment.

## Supplementary Material

Tailored combination prevention packages and PrEP for young key populations
