# Supplementary material for: Tailored combination prevention packages and PrEP for young key populations
Source: J Int AIDS Soc. 2015 Feb 26;18(2Suppl 1):19434. doi: 10.7448/IAS.18.2.19434 (PMC4344537; doi:10.7448/IAS.18.2.19434)
Supplement: Tailored combination prevention packages and PrEP for young key populations [file JIAS-18-19434-s001.pdf]

Supplementary Material

Table 5. Ongoing PrEP studies among young key population, young people, and key populations

**Study name and location**	**Estimated enrollment**	**Age range**	**Include under 18**	**Main study pop-ulation**	**Study objective and design**	**Progress**
ADAPT (HPTN 067)<br><br>South Africa, Thailand, US (Harlem)	360 MSM/ Transgender women<br>180 Women (high risk)	18+	No	MSM (Bangkok, Harlem)<br>Women (Cape Town)	Design: Phase II, Randomized, Open-Label, Pharmacokinetic and Behavioral Study<br>Overview: Identifies PrEP pill-taking schedules that participants are more likely to follow and determines if these schedules influence healthier sexual practices<br>Study arm(s):<br>- Daily: one tablet oral TVF daily regardless of sexual activity<br>- Time driven: one tablet oral TVD 2 days/week and post-exposure booster within 2 hours of sexual intercourse<br>- Event driven: one table oral TVD prior to sexual intercourse and a post exposure booster within 2 hours or sexual intercourse	- Started August 2011<br>- Closed to accrual May 2014
Bangkok Tenofovir Study Open Label Extension<br><br>Thailand	2413	20-60	No	PWID	Open label extension<br>Overview: Follow-on trial of daily oral TDF in PWID	- Started mid- to late-2013<br>- Expected end late-2014

California Collaborative Treatment Group Consortium/ ALERT (CCTG 593)<br><br>US (Long Beach, Los Angeles, San Diego)	600 (testing and linkage)<br>400 (PrEP adherence)	18+	No	MSM	Demonstration project<br>Overview: Determines if those recently screened for HIV will accept assistance to be linked into appropriate health services. Subjects screened for HIV at any CCTG consortium site will be offered linkage to PrEP or care, depending on HIV status. HIV negative participants will also be randomized to receive a text-messaging-based adherence intervention to improve adherence to PrEP.<br>Study arm(s):<br>- link newly diagnosed, HIV infected persons from HIV testing sites to HIV specialist providers<br>- link confirmed HIV negative persons with continued high risk behaviors to PrEP centers<br>- text messaging–based adherence intervention	Linkage interventio n<br>- Started July 2013<br>- Expected end July 2016<br>Adherence interventio n<br>- Started January 2013<br>- Expected end July 2016
CHAMPS-SA Plus Pills<br><br>South Africa	150 (Plus Pills arm)	15-19	Yes	Young men<br>Young women	Demonstration project<br>Overview: Combines HIV prevention strategies into an optimized prevention ´menu´ for adolescents, including PrEP, microbicides, HIV counseling and testing and circumcision.<br>Treatment arms: CHAMPS is comprised of three pilot studies rolled out as a series of interrelated protocols.<br>- Modes of PrEP administration (injectable, oral and vaginal ring) “Uchoose”<br>- Pre-exposure Prophylaxis PrEP “Plus pills”<br>- Male circumcision “MACHO” (Males Actively Choosing Healthy Options)	- Started 2014<br>- Expected end 2015

DemoPrEP<br><br>Brazil	400	18+	No	MSM<br>TGW	Demonstration project<br>Overview: Assesses the acceptability, feasibility and safety of daily, oral TVD as PrEP over 12 months<br>Study arm(s):<br>- TVD	- Started January 2014<br>- Expected end January 2016
The Demo Project (NIAID)<br><br>US (Miami, San Francisco, Washington, DC)	300 (San Francisco)<br>200 (Miami)<br>100 (Washington DC)	~25% ≤ 25	Unknown	MSM<br>TGW	Demonstration project<br>Overview: Determines the level of community interest in PrEP, evaluates adherence to a daily prevention regimen, examines how long they stay on it, and assesses changes to sexual practices<br>Study arm(s):<br>- TVD with regular HIV testing, screening and treatment for other STIs, monitoring for PrEP drug side effects and changes in kidney function, medication adherence and HIV risk-reduction counseling, and free condoms	- Started October 2012.<br>- Expected end August 2014
Durbar (DMSC) and Ashodaya Samithi<br><br>India	2000	18-49	No	TGW<br>FSW	Demonstration project<br>Overview: Assesses the use of a risk assessment tool to identify FSWs who would most benefit from PrEP, collect information about the reasons why FSWs choose to accept or decline PrEP, evaluate two different PrEP delivery strategies (weekly clinic pick-up or home delivery by peer educators every second day), monitor adherence to and discontinuation of PrEP, and evaluate unintended consequences of the use of PrEP in these communities<br>Study arm(s):	- Started November 2014<br>- Expected end October 2016

					- Peer educator deliver TVD every other day<br>- Weekly clinic pick-up of TVD	
East Bay Consortium/ CRUSH<br><br>US (California)	670	18-29	No	YMSM of color<br>TGW<br>Men/ women with HIV positive partner	Demonstration project<br>Overview: Test and link young MSM of color to sexual health services; enhance and evaluate engagement and retention strategies for HIV positive young MSM of color; and engage and retain HIV-negative young MSM of color in sexual health services, including PrEP	- Started December 2013<br>- Expected end January 2017
FACTS 002<br><br>South Africa	100	16-17	Yes	Young women	Phase II Randomized Controlled Trial<br>Overview: Tests the safety and acceptability of tenofovir gel in South African young women<br>Treatment arms:<br>- TDF vaginal gel (BAT 24 dosing; one dose of gel within 12 hours before sex and a second dose of gel as soon as possible within 12 hours after sex and no more than two doses in a 24 hour period)	- Under review by South African Medicines Control Council, conditional on FACTS 001 results
HPTN 073<br><br>US (Chapel Hill, Los Angeles, Washington, D.C.)	225	18+	No	Black MSM	Open label demonstration study with PrEP + C4 model<br>Overview: Assesses initiation, acceptability, safety, and feasibility of PrEP for Black MSM utilizing client-centered care coordination (C4) models<br>Study arm(s):<br>- TVD combined with C4	- Started 2013<br>- Expected end January 2016

IPERGAY<br><br>France, Canada, Germany	1900	18+	No	MSM<br>TGW	Randomized controlled trial<br>Overview: Tests if TVD can offer protection against HIV infection if taken on an intermittent or 'on demand' basis as PrEP<br>Study arm(s):<br>- placebo<br>- on demand TVD (2 pills 2-24 hours before sex, 2 pills after sex (1 pill every 24 hours))	- Started in France January 2012<br>- Started in Quebec July 2013<br>- Start pending in Germany<br>- Expected end December 2016
Los Angeles County PATH PrEP Demo Project<br><br>US (Los Angeles)	375 (PrEP)	18+	No	MSM<br>TGW	Demonstration project<br>Overview: Extensive screening for HIV among populations in high disease burden locations in Los Angeles County. PrEP offered to eligible HIV-uninfected persons. Social network testing intervention among high-risk MSM, with linkage to care for newly diagnosed HIV-positive people. Tests strategies to re-engage out-of-care HIV-infected people using social networks, a peer navigation program and an intensive case management strategy<br>Study arm(s):<br>- TVD	- Started May 2013<br>- Expected end May 2017
LVCT and SWOP<br><br>Kenya	Unknown	Un-known	Unknown	MSM<br>FSW<br>Young women	Demonstration project<br>Overview: Introduces PrEP into combination prevention interventions targeting young women, FSWs, and MSM. Formative	- Formative research in planning

					research underway to assess consumer perceptions and identify potential barriers and opportunities related to introduction. Outcomes include criteria for PrEP indication among young women and a menu of interventions for target populations, including PrEP and feasible delivery options.	phase<br>- Feasibility study report results December 2013
MTN 017<br><br>Peru, South Africa, Thailand, United States, Puerto Rico	186	Un-known	Unknown	MSM<br>TGW	Phase II safety and acceptability trial<br>Overview: Evaluates rectal safety, drug absorption and acceptability of a reduced glycerin formulation of tenofovir gel and oral TVD among MSM and TGW<br>Study arm(s): Participants follow each regimen for 8 weeks with a week break between regimens<br>- Reduced glycerin tenofovir gel used daily and before and after sex<br>- Reduced glycerin tenofovir gel used before and after sex<br>- TVD tablets taken daily	- Started September 2013<br>- Expected end 2015
NEXT-PREP (HPTN 069/ACTG 5305)<br><br>US (Baltimore, Boston, Chapel Hill, Cleveland, Los Angeles,	400 (MSM)<br>200 (women)	18+<br>Sites encouraged to enroll YMSM,MSM and women of	No	MSM<br>Women at risk	Phase II Randomized, Double-Blind, Safety and Tolerability study<br>Overview: Designed to learn more about the safety and acceptability of four different drug combinations when used as PrEP by MSM and women<br>Study arm(s):<br>- Maraviroc<br>- Maraviroc + Emtricitabine<br>- Maraviroc + Tenofovir<br>- Tenofovir + Emtricitabine (TVD)	- Started July 2012

Newark, New York, Philadelphia, Pittsburgh, San Francisco, San Juan, Seattle, Washington, DC)		color				
Partners Demonstration Project<br><br>Kenya, Uganda	1000	18+	No	Serodiscordent couples (mostly young)	Open label demonstration project<br>Overview: PrEP is offered as a 'bridge' until 6 months after ART initiation by the HIV-infected partner	- Started August 2013<br>- Expected end 2016
Project PrEPare (ATN 110)<br><br>US	200	18-22	No	YMSM	Open Label Demonstration Project and Phase II Safety Study<br>Overview: Explores the safety, acceptability and feasibility of PrEP among young men who have sex with men (YMSM) who are at risk for HIV infection<br>Study arms:<br>- 2 Behavioral intervention (Many Men, Many Voices (3MV) and Personalized Cognitive Counseling (PCC)) before administering TVD	- Started November 2012<br>- Expected end November 2015
Project PrEPare (ATN 113)<br><br>US	100	15-17	Yes	YMSM	Open Label Demonstration Project and Phase II Safety Study<br>Overview: Designed to explore the safety, acceptability and feasibility of PrEP among young men who have sex with men (YMSM) who are at risk for HIV infection.	- Started March 2013<br>- Expected end March 2016

					Study arms:<br>- 2 Behavioral intervention (Many Men, Many Voices (3MV) before administering TVD	
PROUD<br><br>UK (London)	500 (546 as of April 2014)	18+	No	MSM	Open label pilot study for Phase IV trial<br>Overview: Assesses trial feasibility, level of interest in PrEP in clinic populations, acceptability of randomization, risk behavior over time (self-report, STIs), change in risk following behavioral interventions, adherence behavior over time, and facilitators and barriers to reducing risk and adhering to a daily pill<br>Study arm(s):<br>- TVD immediately<br>- TVD after 12 months	- Started November 2012<br>- Fully enrolled April 2014<br>- Expected end November 2015
SAPPH-IRe<br><br>Zimbabwe	800	18+	No	FSW	Open label demonstration project of TDF/FTC<br>Overview: The demonstration project is nested within the intervention arm of a cluster randomized trial (anticipated recruitment of 800 FSW). Evaluation will use two population based surveys pre- and post-intervention in the 14 trial sites	- Started
Sibanye Health Project<br><br>South Africa	200	18+	No	MSM	Pilot study<br>Overview: Evaluates the acceptability and uptake of a combination package of biomedical, behavioral and community-level HIV prevention interventions and services for MSM in South Africa<br>Study arm(s):<br>- TVD	- Started May 2014<br>- Expected end April 2015
SPARK Project NYC	445	22-52	No	MSM<br>TGW	Demonstration project<br>Overview: Evaluates a program in which PrEP	- Started January

US (New York)					is introduced, provided, and supported as part of a comprehensive prevention package. Identifies and examines social and behavioral factors associated with disparities in access to prevention and care services among gay, bisexual, and other MSM in NYC that might direct or impact PrEP implementation programs and policies.<br>Study arm(s):<br>- basic PrEP education<br>- sexual health counseling intervention	2014<br>- Expected end July 2017
Sustainable Health center Implementation PrEP Pilot Study (SHIPP) (CDC Foundation)<br><br>US	1200 (total)<br>600 (MSM)	18+	No	MSM<br>Women<br>Men<br>PWID	Health services implementation pilot study<br>Overview: Observational cohort of HIV-uninfected persons receiving daily oral PrEP at four federally qualified health centers that provide sexual health and primary care services to communities with high HIV incidence/prevalence. Studies how to incorporate the delivery of daily oral pre-exposure prophylaxis (PrEP) into the services provided by health centers serving sexually active adults at high risk of acquiring HIV infection.<br>Study arm(s):<br>- TVD	- Started June 2014<br>- Expected end May 2017
VicPrEP Demonstration Project<br><br>Australia	200	Un-known	Unknown	Gay men<br>PWID<br>Sero-discordent couples	Demonstration project<br>Overview: Examines the effectiveness of PrEP in the local settings and the factors contributing to its success<br>Study arm(s):<br>- TVD (participant choice)<br>- no TVD (participant choice)	- Expected start late 2014

TAPS: Expanded use of ART for treatment and prevention for female sex workers in South Africa<br><br>South Africa (Hillbrow, Waterval Boven)	605	18+	No	FSW	Demonstration project<br>Overview: Assesses whether oral PrEP and TasP can be rolled out within a combination prevention and care approach tailored to FSWs	- Started February 2014<br>- Expected end September 2016

MSM = men who have sex with men
TWG = transgender women
FSW = female sex workers
PWID = people who inject drugs
TDF= tenofovir
TVD= emtricitabine/tenofovir (FTC/TDF)
